# Predicting Drug-Target Interactions with Electrotopological State Fingerprints and Amphiphilic Pseudo Amino Acid Composition

**DOI:** 10.3390/ijms21165694

**Published:** 2020-08-08

**Authors:** Cheng Wang, Wenyan Wang, Kun Lu, Jun Zhang, Peng Chen, Bing Wang

**Affiliations:** 1 Department of Computer Science & Technology, Tongji University, Shanghai 201804, China; wangcheng0788@tongji.edu.cn; 2 School of Electrical & Information Engineering, Anhui University of Technology, Ma’anshan 243002, China; wenyanwang9203@gmail.com (W.W.); kunlu0819@gmail.com (K.L.); 3 Key Laboratory of Power Electronics and Motion Control Anhui Education Department, Ma’anshan 243032, China; 4 Institutes of Physical Science and Information Technology & School of Internet, Anhui University, Hefei 230601, China; 00568@ahu.edu.cn

**Keywords:** drug-target interactions, E-state fingerprints, APAAC, distance-based negative sampling, support vector machines

## Abstract

The task of drug-target interaction (DTI) prediction plays important roles in drug development. The experimental methods in DTIs are time-consuming, expensive and challenging. To solve these problems, machine learning-based methods are introduced, which are restricted by effective feature extraction and negative sampling. In this work, features with electrotopological state (E-state) fingerprints for drugs and amphiphilic pseudo amino acid composition (APAAC) for target proteins are tested. E-state fingerprints are extracted based on both molecular electronic and topological features with the same metric. APAAC is an extension of amino acid composition (AAC), which is calculated based on hydrophilic and hydrophobic characters to construct sequence order information. Using the combination of these feature pairs, the prediction model is established by support vector machines. In order to enhance the effectiveness of features, a distance-based negative sampling is proposed to obtain reliable negative samples. It is shown that the prediction results of area under curve for Receiver Operating Characteristic (AUC) are above 98.5% for all the three datasets in this work. The comparison of state-of-the-art methods demonstrates the effectiveness and efficiency of proposed method, which will be helpful for further drug development.

## 1. Introduction

Drug-target interaction (DTI) prediction is of great significance for pharmacology development [1,2]. The problem can be solved by experimental ways. However, due to the lack of relevant theoretical knowledge, experimental methods are easy to get a high failure rate, and are restricted by their high economic and time cost [3,4]. According to reports, it often takes decades for a new drug to be approved by US Food and Drug Administration (FDA) [5]. With the improvement of a relevant knowledge system, the hypothesis that a single drug corresponds to a single target has been extended, which makes the original DTI problems more complex [6]. Therefore, computational methods have attracted more attention in DTI research in recent years [4,7,8,9].

The computational methods can be summarized as docking simulation and machine learning-based methods. Docking simulation is the most successful method in drug-target interaction prediction when a three dimensional native structure of the target protein is available [10]. However, it is a time-consuming and expensive process to determine the native structure of a protein by sophisticated methods like X-ray Crystallography [11]. Thus, the 3D-structure of proteins are often unavailable. Therefore, researchers are encouraged to apply machine learning-based methods to tackle the problems by formulating DTIs as supervised learning models. It largely depends on the training dataset composed with reliable positive and negative samples in supervised learning [12]. Yamanishi et al. proposed gold standard datasets for supervised learning methods with four sets of target proteins and drugs based on KEGG BRITE, BRENDA, SuperTarget and DrugBank [13]. These subdatasets have been used in a large number of related papers [14,15,16,17,18,19]. Due to the small number of known validated interactions among drug-target pairs, the unlabeled interaction pairs are considered as negative samples in most research and thus they outnumber positive samples [6,11,20,21,22]. The imbalance of DTI datasets is a major problem in supervised learning [11,23]. Another challenge is the representation of the drug-target pair which is often generated from molecular fingerprints of drugs and sequence or structure-based information of proteins [11].

Similarity-based and feature vector-based approaches are the main two aspects to solve DTI problems in supervised learning [16,24]. Similarity-based methods were proposed based on the assumption that “similar drugs share with similar targets” [16]. Yamanishi et al. proposed a pharmacological space for DTI predictions based on similar structures, and first introduced a gold standard dataset which has been exhaustively used by researchers [13]. PDTD (Predicting Drugs Targets with Domains) made an appealing hypothesis that a similar domain reflects similar therapeutic effect and protein domain was suggested as a powerful piece of information to discriminate drug-target interactions [25]. Liu et al. considered the similarity of drug side-effects, protein-domain and function annotation semantic of targets to predict DTIs [26]. Lan et al. added similar 3D-structures of proteins and calculation based on GO term to screen the available drug-target pairs [27]. More details of DTI research based on similarity can be found in the review written by Ozturk et al. [16]. Although similarity-based methods have good effects on predictions of DTI, they are also restricted in the case of less information with drug or target protein interactions. Feature vector-based methods can combine drug-descriptors with target-descriptors to solve these problems. Wei et al. combined 881-dimensional drug-descriptors from Pubchem, with 567-dimensional and 1449-dimensional target-descriptors from protein sequences to predict DTIs [28,29]. The 1024-dimensional drug-descriptor extracted by PaDEL-descriptor, and the 1287-dimensional target-descriptor calculated based on amino acid composition were used for predictions of DTI [15,30]. The 193-dimensional drug-descriptor developed based on Rcpi package is combined with the 1290-dimensional target-descriptors extracted from PROFEAT for DTI predictions [7,31]. The commonly used classification models, such as support vector machines (SVM), random forest, random walk with restart, and decision trees can be found both in similarity-based and feature vector-based algorithms [8,14,15,32,33,34]. With the expansion of data scale, deep learning is widely used in DTIs [11,35].

Deep learning-based methods have addressed many biological issues especially on large scale datasets [36,37,38]. Deep-Belief Network (DBN) was proposed by Feng et al. to predict DTIs, which is based on 6144-dimensional Extended-Connectivity Fingerprints (ECFP) of drugs, and 8420-dimensional Protein Sequence Composition (PSC) of target proteins [39]. Wen et al. constructed a novel predictor called DeepDTIs to predict possible interactions between drugs and targets [38]. Farshid et al. proposed FRnet-Encode to extract 4096 features, and FRnet-Predict to predict drug-target interactions based on deep convolutional neural network [11]. Hu et al. introduced a convolutional neural network system to discriminate drug-target interactions [35]. A large number of hyper-parameters adjustment is the key factor restricting the application of deep learning. Another challenge in DTIs is the increasing dimension of features, even in deep learning.

In this work, an effective and efficient representation of drug-target pairs is introduced to infer possible DTIs. The drug-descriptors are encoded based on the electrotopological state (E-state) of molecules, which can be extracted by PaDEL-Descriptor [30,40]. The target-descriptors, called amphiphilic pseudo amino acid composition (APAAC), was first introduced by Chou, and it can be generated by the webservice on PROFEAT [41,42,43]. For drugs and target proteins, the dimensions of these two descriptors are only 79 and 80, respectively. The gold standard datasets introduced by Yamanishi et al. is used for training and testing in this work. To solve the imbalance problem of datasets, we calculated the Euclidean distance between unlabeled drug-target pairs and positive ones, which is called distance-based sampling for negative DTIs [35]. At last, SVM is used for classification with less parameter adjustment [33,44]. The experiments are executed 100 times and the average results are shown. To further demonstrate the effectiveness of proposed methods, external independent datasets are also tested. As a result, our work outperforms most state-of-the-art methods on the same benchmark datasets.

## 2. Results

### 2.1. Performance Evaluation on DTIs

In the experiments reported in this paper, the gold standard dataset is used as benchmark for training and testing. The dataset was first introduced by Yamanishi et al., and can be divided into four subdatasets named by the enzyme, G-protein coupled receptors (GPCRs), ion channel and nuclear receptors [13]. Each subdataset is split into two parts—train set and test set, with the ratio of 0.8 and 0.2, respectively. The train and test set are constructed with validated positive samples, and negative samples calculated based on Euclidean distance. In addition, 5-fold cross validation is used to avoid overfitting, i.e., each fold of each subdataset is tested once [45,46]. To obtain stable and reliable results, all experiments are executed 100 times, and the average results are shown. The drug-descriptors, E-state fingerprints, are extracted by PaDEL-Descriptor, which is a free software for compound descriptors generation [30]. PROFEAT is a webservice for calculating protein features, which is used in this paper for APAAC [41,42]. The dimensions of features used in this work are 79 and 80 for drugs and targets, respectively.

Various performance criteria are available for drug-target interaction predictions. The measurement of Accuracy is one of them, which shows that the accurate percentage of the model, is sufficient in general, but holds no significance for imbalance datasets [11]. Some criteria such as Recall, Precision and F1-scores, are also affected by the ratio of imbalance datasets. The metric, namely area under curve for Receiver Operating Characteristic (AUC), is independent from the ratio of the dataset, and is widely used in most research as standard criteria for comparison [11,21]. The range of AUC is from 0 to 1, and the higher the value, the more effective the model is. When a random classification occurs, the AUC is equal to 0.5. In this work, AUC is the main metric for model evaluation. In addition, we extract the same number of negative samples as positive ones to make more evaluation criteria feasible. The metrics used in this work except AUC are formulated as follows:

(1) $$\mathrm{Acc}.=\frac{\mathrm{TP}+\mathrm{TN}}{\mathrm{TP}+\mathrm{TN}+\mathrm{FP}+\mathrm{FN}}$$

(2) $$\mathrm{Rec}.=\frac{\mathrm{TP}}{\mathrm{TP}+\mathrm{FN}}$$

(3) $$\mathrm{Prec}.=\frac{\mathrm{TP}}{\mathrm{TP}+\mathrm{FP}}$$

(4) $$\mathrm{MCC}=\frac{\mathrm{TP}\times \mathrm{TN}-\mathrm{FP}\times \mathrm{FN}}{\sqrt{\left(\mathrm{TP}+\mathrm{FP}\right)\left(\mathrm{TP}+\mathrm{FN}\right)\left(\mathrm{TN}+\mathrm{FP}\right)\left(\mathrm{TN}+\mathrm{FN}\right)}}$$

(5) $$F1\_score=\frac{2\times precision\times recall}{precision+recall}$$

where the True Positive (TP) is the number of drug-target pairs predicted as interactions correctly; the False Positive (FP) indicates the number of negative drug-target pairs classified as interactions incorrectly; True Negative (TN) represents the number of negative pairs predicted as non-interactions correctly; False Negative (FN) is the number of positive pairs classified as non-interactions incorrectly [47]. Acc., Rec., Prec., F1. and MCC are the short for Accuracy, Recall, Precision, F1-scores and Matthews Correlation Coefficient, respectively [47].

**Figure 1:**
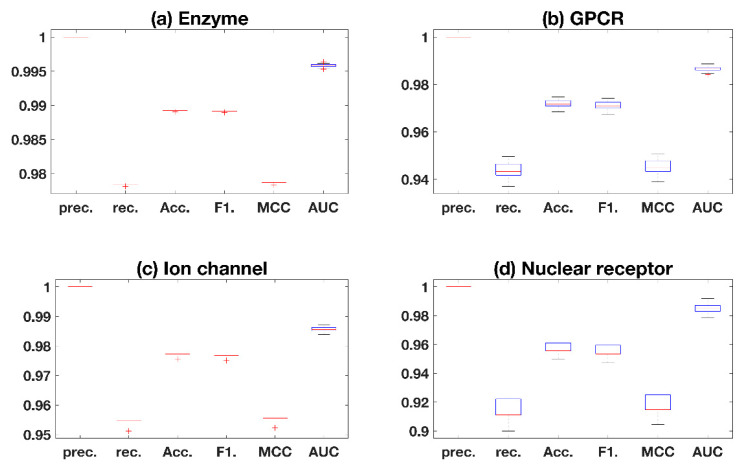
Distribution of results in benchmark datasets. Prec., rec., acc., F1. and MCC are abbreviations of Precision, Recall, Accuracy, F1-scores and Matthews Correlation Coefficient, respectively. AUC indicates the area under ROC-curve.

**Figure 2:**
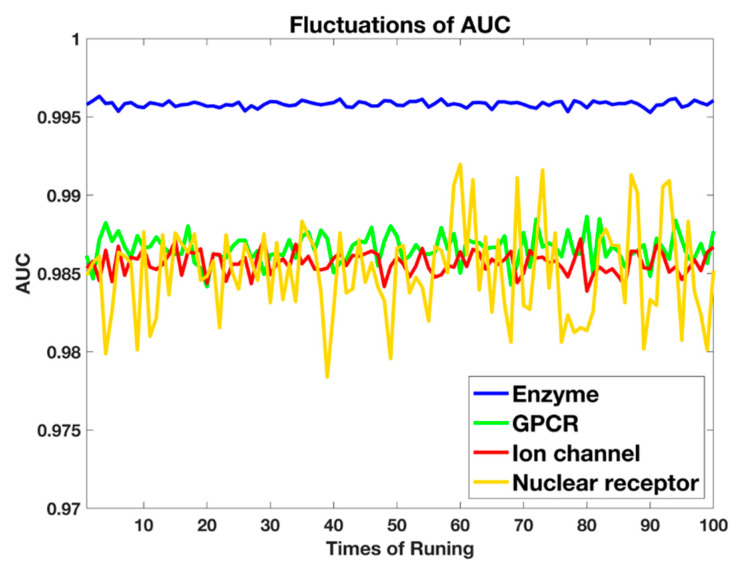
The fluctuations of AUC values in four subdatasets after executing 100 times.

The average prediction performance is listed in Table 1, with the representation value of average ± standard deviation. From Table 1, the results show that excellent precision values are obtained, which means non-interaction pairs are not mispredicted. In addition, the good recall values indicate that the model has less false negative samples. All of the four AUC values in subdatasets are higher than 98.5%, which demonstrate the great prediction and the effectiveness of our proposed method. The standard deviation value also shows the robustness of proposed work, which is important for further generalization, as shown in Figure 1. To represent the stability of key measurement of the AUC value clearly, Figure 2 plots the fluctuations with 100 times repeated experiments. It is shown that poor robustness is obtained in nuclear receptors, compared with other three subdatasets. This may be caused by the small size of nuclear receptors, which makes the training insufficient to obtain stable models [11].

### 2.2. Comparison with State-of-the-Art Methods

Various algorithms based on the same gold standard datasets are compared. To make the comparison comprehensive, we choose methods based on both similarity and feature vector. Four similarity-based methods, Bipartite Graph Learning (Bigram), KBMF2K, NetCBP and PUDT are compared [6,8,22,27]. The feature vector-based methods of Wang et al., MFDR, Cao et al. and FRnet-DTI, are compared with our work [5,11,28,48]. Considering that different negative sampling methods will affect the final results of predictions, we use random sampling for negative DTIs to test, just like other methods based on feature vectors, to ensure the comparison is reliable. The results of these comparative methods are taken from their papers. AUC is chosen as the unified measurement of the comparison, as shown in Table 2. Our methods with random and distance-based sampling for negative DTIs are marked as Ran-proposed and Dis-proposed, respectively. Although the performance of Ran-proposed is not better than some of methods in Table 2, it is close to the best one of FRnet-DTI. Moreover, compared with 4096 dimensions used in FRnet-DTI, Ran-proposed only used 159-dimensional features to get closed results. That demonstrates the effectiveness of proposed features in this work are within a lower computation cost. It can be seen from Table 2 that the results of Dis-proposed are the best in enzymes, GPCRs, ion channel and nuclear receptors, which are 0.996, 0.987, 0.986 and 0.985, respectively. Furthermore, the comparison between Ran-proposed and Dis-proposed shows that the distance-based sampling for negative DTIs is more effective than random sampling. It is interesting to notice that most results based on feature vectors have poor predictions in nuclear receptors, compared with other three subdatasets. The problem may be due to it having the smallest size of dataset, which makes the training of the model insufficient, but this is not obvious in our distance-based sampling method. Our method is shown effective by the results, even in small datasets.

The external validation dataset is also used to prove the effectiveness of the proposed method. Distance-based sampling for negative DTIs was first used in Hu et al., whose training sets were composed of reference [1] and manually collected datasets [35]. The replication of the algorithm of Hu et al. is difficult without their original dataset. Therefore, another algorithm, namely DeepDTI and their dataset, which is also compared in Hu et al., is chosen for further comparison and validation of our method. To make the comparison reliable, random sampling for negative DTIs, as same as in DeepDTI, is used with the proposed features, which is marked as Ran-proposed. Meanwhile, Dis-proposed represents the method with distance-based sampling for negative DTIs. The results and their feature dimensions used are shown in Table 3. The True Positive Ratio (TPR) is defined as TP/(TP + FN), which is equal to Recall, and the True Negative Ratio (TNR) is defined as TN/(TN + FP). It is shown that the performance of Ran-proposed is slightly lower than that of the DeepDTI without considering the feature dimension. At the same time, the feature dimension of Ran-proposed is only 159, which is obviously smaller than 14,564 used in DeepDTI. In addition, compared with DeepDTI, Ran-proposed are only 0.6%, 7.82%, 3.19% and 2.54% worse on TPR, TNR, Accuracy and AUC, respectively. In summary, Ran-proposed can still be considered a good predictor with its low complexity. The superior performance of Dis-proposed is shown in Table 3, and its standard deviations of TPR, TNR, Accuracy and AUC are 0.3%, 1.24%, 0.35% and 0.55% smaller than DeepDTI, respectively, which proves the effectiveness and robustness of our work. In addition, the comparison results of DeepDTI in Hu et al. are also shown in Table 3. It is shown that our work is superior than Hu et al. with the same method of negative sampling.

To further demonstrate the effectiveness of our method, the independent dataset extracted from ChEMBL is tested [49]. The dataset is extracted from Drug Mechanism of ChEMBL, retaining the inhibitors and Homo sapiens. In this study, a total of 1928 drug-target interaction pairs are obtained, which consisted of 1304 drugs and 682 targets. The repeated interactions in this study are eliminated. In addition, we also test different negative sampling methods, that are random sampling and distance-based sampling, which are marked as Ran-ChEMBL and Dis-ChEMBL, respectively. As shown in Table 4, the performance of our method is satisfactory, which further proves the effectiveness and lack of overfitting of the proposed work.

## 3. Discussion

### 3.1. Effectiveness of Negative Generation

Two different methods for negative sampling are compared and tested in this work. Considering the imbalance of the DTI dataset, most researchers select negative samples randomly from unlabeled drug-target pairs, which is random sampling. Although the method has good prediction results in various papers, there is still some shortcomings, such as difficulty in replication and ease for production of incorrect negative samples. Hu et al. proposed a new method for negative sampling based on Euclidean distance calculation, called distance-based sampling in this work. This method combines drug and target-descriptors as a specific space, and all interactions and non-interactions are regarded as nodes in this space. The Euclidean distance between each unlabeled drug-target pair and the positive center is calculated, where the positive center is defined as the mean vector of all the positive samples, which will be discussed later. The distance between two drug-target pairs is considered as a measurement of their similarity, that is, the greater the distance, the less the similarity [35]. At the same time, all unlabeled drug-target pairs are sorted according to the distances calculated, the less the similarity, the greater the possibility of negative samples. Therefore, more reliable negative samples are screened out than random sampling.

**Figure 3:**
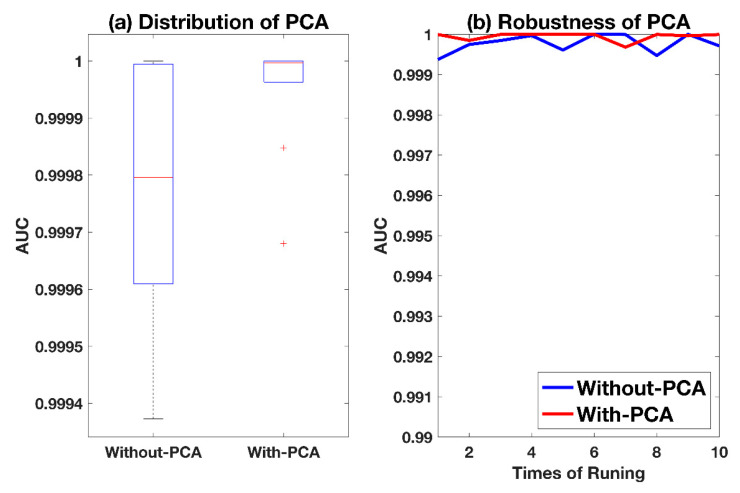
Comparison between different methods for the calculation of the positive center. With-PCA and Without-PCA represent the calculation with and without PCA, respectively. (**a**) Plot of the distribution of the two methods. (**b**) Plot of the trend of the two methods after 10 repetitions.

The method of obtaining the center of positive samples is discussed by experiment. Considering the implicit correlation of each dimension of drug-target pairs, the principal component analysis (PCA) is used firstly, and then the mean value of these orthogonal vectors is calculated as the positive center [50]. In this work, the results with PCA processing is marked as With-PCA, and the method of only calculating the mean of original features without PCA is marked as Without-PCA. The experimental results of With-PCA and Without-PCA are based on datasets in DeepDTI, which are represented with the AUC in Figure 3. More reliable results are obtained based on With-PCA, with a standard deviation of 0.1%. For the results of Without-PCA, its standard deviation is only 0.1% lower than that of With-PCA in Figure 3a, and the average results of 10-time repetition, which are shown in Figure 3b, are very close. Considering the effect is comprehensive, Without-PCA, with good results and low complexity, is selected in this work.

**Figure 4:**
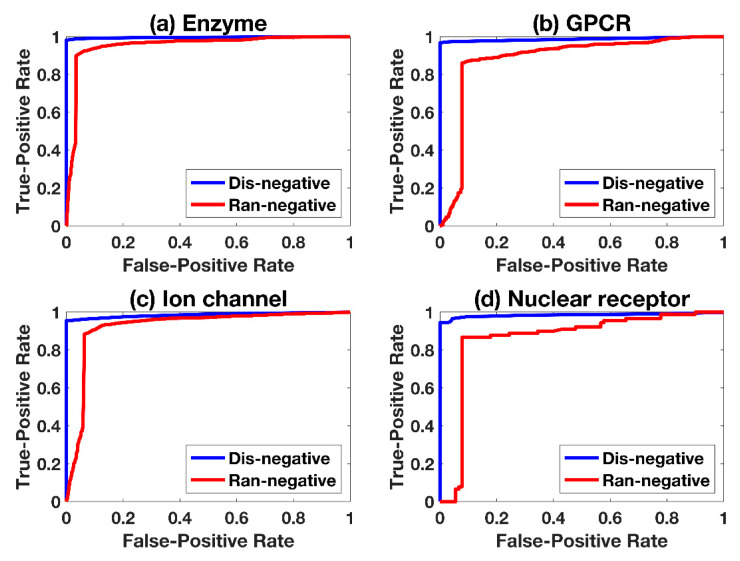
ROC-curves of two negative sampling methods. Ran-negative and Dis-negative represent the setting of negative sampling based on random and distance, respectively.

**Figure 5:**
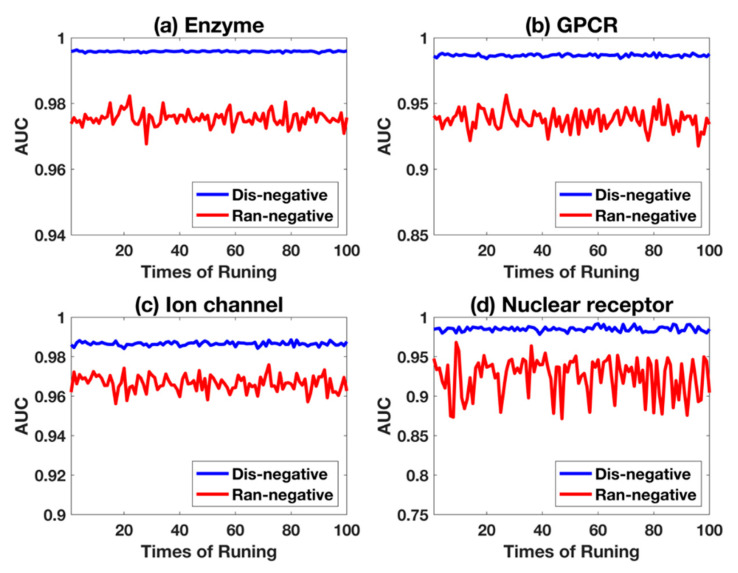
The robustness of two negative sampling methods. Ran-negative and Dis-negative indicate the experimental setting for negative sampling based on random and distance in 100-times repetition, respectively.

Comparison between random and distance-based sampling for negative samples is shown in Figure 4, where Ran-negative and Dis-negative represent the experimental setting of negative sampling with random-based and distance-based, respectively. The receive operating characteristic curves (ROC-curves) show clearly that Dis-negative is superior than Ran-negative, and their robustness is shown in Figure 5 with 100-times repetition. Ran-negative shows larger fluctuations, indicating that the performance of Dis-negative used in this work is more stable.

### 3.2. Discussion of E-State and APAAC

Electrotopological state fingerprints (E-state) and Amphiphilic Pseudo Amino Acid Composition (APAAC) are for the first time being combined in drug-target interactions. Compound descriptors were always extracted based on their structure, substructure, or electronic characteristics. Even if some indexes calculated both the topological structure and electronic features at the same time, different measurements and metrics were used. The E-state indexes were first constructed by Lowell et al., which were derived from counts of electrons within the hybridization model of covalent binding, and were the adjacency relations in the hydrogen-suppressed graph [40]. Furthermore, it was also the first atom-level index which can combine both electronic structures and molecular topology into a single index by using the same metric [40]. Given the results shown in this work, electronic structures and molecular topology may both be important for drug-target interactions. For target proteins, APAAC was proposed by Chou to reflect the sequence-order information, and consider hydrophobicity and hydrophilicity of the constituent amino acids, which plays a very important role in protein folding, and its interaction with environment [43]. It can be regarded as an extension of Amino Acid Composition, adding more sequence-order information. In addition, APAAC had been successful applied in protein representation for the prediction of enzyme subfamily, structure and interactions [43]. Our works extract these two feature vectors, train and test them in gold standard dataset, and obtain good results. The proposed method is helpful for further predictions of drug-target interactions with low complexity. Meanwhile, the relationship among drug-target interactions, electronic structures and molecular topology, protein folding and sequence-order information will be further explored to find out the essence features of DTIs.

### 3.3. Parameters Adjustment of Algorithm

The parameters adjustment of the algorithm is based on the measurements of AUC and their robustness results in 100 repetitions. In this work, the tool of SVM, libsvm v3.23 is used to train, validate and test [44]. According to other DTI methods based on SVM, the radial basis function (RBF) kernel is selected. The kernel only needs to adjust two parameters of *c* and *γ.* The process of adjustment is completed by grid search, that is to fix one parameter and change the other. The adjustment of the two parameters is in the form of exponent, with the bottom of two, and the index ranging from −10 to 10 with step of one [51,52]. At last, the best performance is obtained when *c* = 4 and *γ* = 0.25.

## 4. Materials and Methods

### 4.1. Benchmark Datasets

The gold standard dataset is used to train and test in this work, which was firstly constructed by from KEGG BRITE, BRENDA, SuperTarget and DrugBank by Yamanishi et al. [13]. The dataset is divided into four subdatasets, i.e., enzymes, G-protein coupled receptors (GPCRs), ion channels and nuclear receptors, based on different characteristics of target proteins. The gold standard dataset is publicly available, and its statistics of datasets are shown in Table 5 [13]. The imbalance of datasets can be reflected by the proportion of positive samples, which are only 0.99%, 3.00%, 3.45% and 6.41% in enzymes, GPCRs, ion channels and nuclear receptors, respectively.

The dataset used in DeepDTI (Wen et al.), which is extracted from DrugBank (https://www.drugbank.ca/, the Governors of the University of Alberta, Edmonton, AB, Canada), is for further demonstration of effectiveness of our work [35,38]. There are 6262 drug-target pairs which are validated as positive samples among the whole dataset composed of 1412 drugs and 1520 targets. Considering that the negative samples were generated by random sampling in DeepDTI, we also use random sampling to conduct experiments to ensure the reliability of the comparison. In addition, the proposed work with random sampling and distance-based sampling are marked as Ran-proposed and Dis-proposed for short in Table 3, respectively.

It is extracted from Drug Mechanism of ChEMBL (https://www.ebi.ac.uk/, European Molecular Biology Laboratory (EMBL), Cambridge, UK) to form an independent dataset [49]. The action type of inhibitors, and interactions related with Homo sapiens are retained. At the same time, the interactions which have been recorded in the gold standard dataset are removed. At last, a total of 1928 drug-target interaction pairs are obtained, which consisted of 1304 drugs and 682 targets. The random sampling is also tested and shown in Table 4.

### 4.2. Descriptors of Drugs and Targets

In this work, the electrotopological state (E-state) fingerprints, which are generated from both electronic and topological characters of chemical molecules, are adopted as a drug-descriptor for the prediction of drug-target interactions [40]. The E-state indexes were first constructed by Lowell et al., which were derived from counts of electrons within the hybridization model of covalent binding and the adjacency relations in the hydrogen-suppressed graph [40]. In addition, it was the first time that electronic structure and molecular topology were combined using the same metric [40]. In this work, E-state fingerprints are extracted by PaDEL-Descriptor (version 2.21, Yap Chun Wei, Pharmaceutical Data Exploration Laboratory, Singapore), which is a public software for calculating molecular descriptors and fingerprints [30]. There are 797 descriptors and 10 types of fingerprints that can be achieved in the software [30]. E-state fingerprints are 79-dimensional binary feature vectors with default parameters setting. The value marked with one indicates the drug has the corresponding characteristic, otherwise it is marked as zero.

The target proteins are represented by amphiphilic pseudo amino acid composition (APAAC), which was first proposed by Chou [43]. It is represented to effectively reflect the sequence-order information, and consider hydrophobicity and hydrophilicity of the constituent amino acids, which plays a very important role in protein folding, and its interaction with environment [43]. APAAC is considered effective in drug-target interactions, due to its successful application in protein representation for the prediction of enzymes subfamily, structure and interactions [43]. PROFEAT is a web server used to calculate commonly used structural and physicochemical features of proteins and peptides from amino acid sequences, and is used in this work for calculation of APAAC. The dimension of APAAC is 80, which is extracted by default parameters in PROFEAT [31,41].

### 4.3. Construction of Datasets and Algorithm

Negative instances are generated based on distance-based sampling from the unlabeled drug-target pairs. The center of positive samples is defined as the mean value of all interaction pairs in each dataset. In addition, the Euclidean distance from all the unlabeled samples to the positive center is calculated and sorted. The farther the distance is, the more reliable the sample is to be negative. At the same time, the number of negative samples should equal to the size of positive ones, which will make supervised learning more accurate.

Each subdataset is split into two sets, train set and test set using 5-fold cross validation, with a proportion of 80% and 20%, respectively. Moreover, each experiment is executed 100 times and the average results are considered.

Support vector machines (SVM) and its toolbox Libsvm (version 3.23, Chih-Chung Chang and Chih-Jen Lin, National Taiwan University, Taipei, China) are adopted in this paper [44]. The radial basis function (RBF) kernel is selected for model construction, and two parameters, *c* = 4 and *γ* = 0.25, are obtained and optimized based on grid search.

### 4.4. The Flowchart

**Figure 6:**
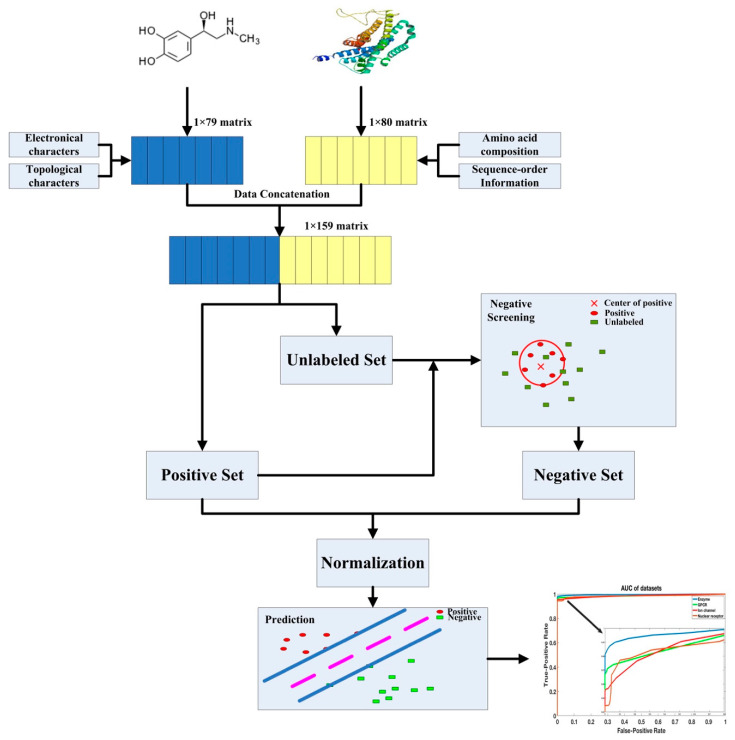
The flowchart of proposed method.

The flowchart is shown in Figure 6, which represents the detail of our proposed method. As shown in Figure 6, firstly, the feature vectors of drugs and targets are extracted and combined to form the feature matrix of positive and unlabeled DT pairs. Then, negative samples are extracted based on the positive center and unlabeled dataset. Finally, SVM is used for training and testing to obtain better models.

## 5. Conclusions

In this paper, low-dimensional features based on E-state fingerprints and APAAC are tested, and satisfactory results are obtained. E-state fingerprints are calculated based on electronic structure and molecular topology with the same metric. Compared with amino acid composition (AAC), APAAC takes into account the sequence-order information of amino acid sequences.

Moreover, the distance-based sampling for negative DTIs is introduced and compared with random sampling. The experimental results show the effectiveness of the proposed features and method. Meanwhile, our method is further demonstrated by another two independent datasets. We believe that the excellent performance of our method will motivate other researchers to explore the potential relationship between features and drug-target interactions.

## Figures and Tables

**Table 1:** Results of proposed method. Prec., Rec., Acc., F1., MCC and AUC are short for precision, recall, accuracy, F1-scores, Matthews Correlation Coefficient and Area Under ROC-curves.

	Enzyme	GPCR	Ion Channel	Nuclear Receptor
Prec. (%)	100.00 ± 0.00 ^#^	100.0 ± 0.00	100.0 ± 0.00	100.0 ± 0.00
Rec. (%)	97.85 ± 0.01	94.38 ± 0.28	95.46 ± 0.03	91.50 ± 0.68
Acc. (%)	98.92 ± 0.01	97.19 ± 0.14	97.73 ± 0.02	95.75 ± 0.34
F1. (%)	98.91 ± 0.01	97.11 ± 0.15	97.68 ± 0.02	95.56 ± 0.37
MCC (%)	97.87 ± 0.01	94.53 ± 0.27	95.56 ± 0.03	91.83 ± 0.63
AUC (%)	99.58 ± 0.02	98.66 ± 0.09	98.57 ± 0.07	98.51 ± 0.30

^#^ The value in the table means that average value ± standard deviation.

**Table 2:** Comparison for state-of-the-art methods on gold standard datasets. Ran-proposed and Dis-proposed represent that the methods with random and distance-based sampling for negative DTIs, respectively.

AUC		Enzyme	GPCR	Ion Channel	Nuclear Receptor	Dimension of Features
Similarity-based	KBMF2K	0.832	0.857	0.799	0.824	-
	NetCBP	0.825	0.823	0.803	0.839	-
	Bigram	0.948	0.872	0.889	0.869	-
	PUDT	0.884	0.878	0.831	0.885	-
Feature vector-based	Cao et al.	0.948	0.890	0.872	0.878	343
	Wang et al.	0.943	0.874	0.911	0.818	1281
	MFDR	0.969	0.904	0.933	0.886	1448/2330
	FRnet-DTI	0.976	0.948	0.951	0.924	4096
	Ran-proposed	0.973	0.926	0.967	0.928	159
	Dis-proposed	0.996	0.987	0.986	0.985	159

**Table 3:** Comparison with DeepDTI and Hu et al. Ran-proposed and Dis-proposed represent the methods based on random sampling and distance-based sampling for negative DTIs. TPR, TNR, Acc. and AUC represent True Positive Ratio, True Negative Ratio, Accuracy and Area Under ROC-curves.

Methods	TPR (%)	TNR (%)	Acc. (%)	AUC (%)
DeepDTI	82.27 ± 0.65 ^#^	89.53 ±1.30	85.88 ± 0.49	91.58 ± 0.59
Hu et al. of Random sampling	91.94 ± 0.91	91.14 ± 1.96	88.14 ± 0.75	95.27 ± 0.43
Hu et al. of Distance-based sampling	97.09 ± 0.67	96.86 ± 1.29	96.04 ± 0.32	99.47 ± 0.21
Ran-proposed	81.67 ± 2.33	81.71 ± 2.51	81.69 ± 1.72	89.05 ± 1.30
Dis-proposed	99.80 ± 0.30	99.97 ± 0.06	99.89 ± 0.14	99.98 ± 0.04

^#^ The value in the table means that average value ± standard deviation.

**Table 4:** Results of the Independent dataset from ChEMBL. Ran-ChEMBL and Dis-ChEMBL represent the experiments with random sampling and distance-based sampling for negative DTIs, respectively.

Methods	Prec. (%)	Rec. (%)	Acc. (%)	F1. (%)	MCC (%)	AUC (%)
Ran-ChEMBL	72.48 ± 4.39 ^#^	90.14 ± 1.22	77.68 ± 3.77	80.23 ± 2.75	57.34 ± 6.68	92.05 ± 1.35
Dis-ChEMBL	99.86 ± 0.24	98.99 ± 0.02	99.41 ± 0.13	99.42 ± 0.12	98.86 ± 0.25	99.83 ± 0.02

^#^ The value in the table means average value ± standard deviation.

**Table 5:** Statistics of gold standard datasets.

	Enzyme	GPCR	Ion Channel	Nuclear Receptor
Drugs	445	223	210	54
Targets	664	95	204	26
Positive Interactions	2926	635	1476	90
Total DT-pairs	295,480	21,180	42,840	1404
proportion of positive	0.99%	3.00%	3.45%	6.41%
